# Enhancing crop resilience to combined abiotic and biotic stress through the dissection of physiological and molecular crosstalk

**DOI:** 10.3389/fpls.2014.00207

**Published:** 2014-05-19

**Authors:** Christos Kissoudis, Clemens van de Wiel, Richard G. F. Visser, Gerard van der Linden

**Affiliations:** Laboratory of Plant Breeding, Wageningen UniversityWageningen, Netherlands

**Keywords:** salinity, drought, disease resistance, R-genes, crosstalk, hormones, transcription factors, post-translational modifications

## Abstract

Plants growing in their natural habitats are often challenged simultaneously by multiple stress factors, both abiotic and biotic. Research has so far been limited to responses to individual stresses, and understanding of adaptation to combinatorial stress is limited, but indicative of non-additive interactions. Omics data analysis and functional characterization of individual genes has revealed a convergence of signaling pathways for abiotic and biotic stress adaptation. Taking into account that most data originate from imposition of individual stress factors, this review summarizes these findings in a physiological context, following the pathogenesis timeline and highlighting potential differential interactions occurring between abiotic and biotic stress signaling across the different cellular compartments and at the whole plant level. Potential effects of abiotic stress on resistance components such as extracellular receptor proteins, R-genes and systemic acquired resistance will be elaborated, as well as crosstalk at the levels of hormone, reactive oxygen species, and redox signaling. Breeding targets and strategies are proposed focusing on either manipulation and deployment of individual common regulators such as transcription factors or pyramiding of non- (negatively) interacting components such as R-genes with abiotic stress resistance genes. We propose that dissection of broad spectrum stress tolerance conferred by priming chemicals may provide an insight on stress cross regulation and additional candidate genes for improving crop performance under combined stress. Validation of the proposed strategies in lab and field experiments is a first step toward the goal of achieving tolerance to combinatorial stress in crops.

## INTRODUCTION

Plants are sessile and cannot escape stressful conditions originating from the physical environment (abiotic stress) and from interactions with insects and microorganisms such as fungi and bacteria (biotic stress). The on-going change in climate conditions due to mostly anthropogenic causes such as the increase in CO_2_ emissions (Peters et al., 2011) exaggerates agricultural land deterioration due to temperature rise. This results in increased evapotranspiration, intensifying drought episodes (Zhao and Running, 2010) and increasing soil salinization, augmenting the 7% of the total and 30% of the irrigated agricultural land already affected by salinity (Munns and Tester, 2008). Available data and projections on the effect of climate change on pathogen spread are not conclusive, although the evidence points to increased reproductive potential and geographic expansion that will lead to interactions with both more hosts and different pathogen strains, increasing the chances for the rise of more virulent strains (Garrett et al., 2006). Therefore, the chances of plants encountering abiotic and/or biotic stress in the future are likely to be higher, with more frequent stress interactions.

Plants have developed a multitude of defense responses that allow them to adapt, survive and reproduce under stress conditions (Pieterse et al., 2009). With the advancement of *~omics* technologies and on-going functional characterizations of individual genes, it has become apparent that environmental adaptation is under tight regulation, which is critical for plant survival (López et al., 2008). Many components of this regulatory network are involved in responses to different stresses but may function antagonistically or some responses are prioritized over others, compromising plant resistance to multiple stresses simultaneously (Glazebrook, 2005; Yasuda et al., 2008).

Major components of the regulatory networks underlying environmental stress adaptation, pathogen recognition, and defense include reactive oxygen species (ROS) signaling (Miller et al., 2008), plant hormones (Bari and Jones, 2009; Peleg and Blumwald, 2011), changes in redox status (Munne-Bosch et al., 2013), and inorganic ion fluxes, such as Ca^2+^ (Martí et al., 2013). Based on *~omics* data analyses these components appear to be at least partly shared between both abiotic and biotic stress signaling, indicating crosstalk and convergence of mechanisms in these pathways and the existence of a general stress response (Walley et al., 2007).

The nature of pathogen perception dictates that physical barriers such as the cuticle, stomata, and cell walls are also critical for timely pathogen recognition and interception (Asselbergh et al., 2007). As data generated by ~*omics* analyses derive from a mixture of different cell types and tissues, these spatially important interactions may be missed and these datasets may lead to erroneous conclusions about components shared and their significance in abiotic and biotic stress crosstalk. Moreover, as combinatorial stress potentially results in novel interactions between signaling components, extrapolation of results from studies with single stress conditions should be done with care.

Here we will elaborate on the mechanisms involved in adaptation and tolerance to combinatorial abiotic and biotic stress, with a focus on dehydration/salt stress and fungal and bacterial pathogens interaction. This review will particularly emphasize interactions that potentially arise during the pathogenesis timeline and were as yet given little attention. We will discuss molecular components with potentially critical roles in abiotic and biotic stress tolerance crosstalk, and propose breeding approaches toward effective crop improvement against combinatorial stress.

## EVIDENCE OF CROSSTALK

### EVIDENCE AT THE PHENOTYPIC AND PHYSIOLOGICAL LEVEL

Studies on the commonly occurring combination of drought and heat stress have revealed that physiological and molecular responses of plants exposed to both stresses are markedly different from their response to the individual stresses (Rizhsky et al., 2004). Similarly, there are numerous reports about abiotic stress (mostly drought and salinity) affecting pathogen resistance, which is indicative of interaction between abiotic and biotic stress. There are reports of disease resistance attenuation by high humidity and high temperature (Wang et al., 2005, 2009). In most cases, abiotic stress predisposes plants to subsequent pathogen infection (Sanogo, 2004; Triky-Dotan et al., 2005; You et al., 2011), although positive effects on resistance to foliar pathogens have also been reported (Wiese et al., 2004; Achuo et al., 2006).

**FIGURE 1:**
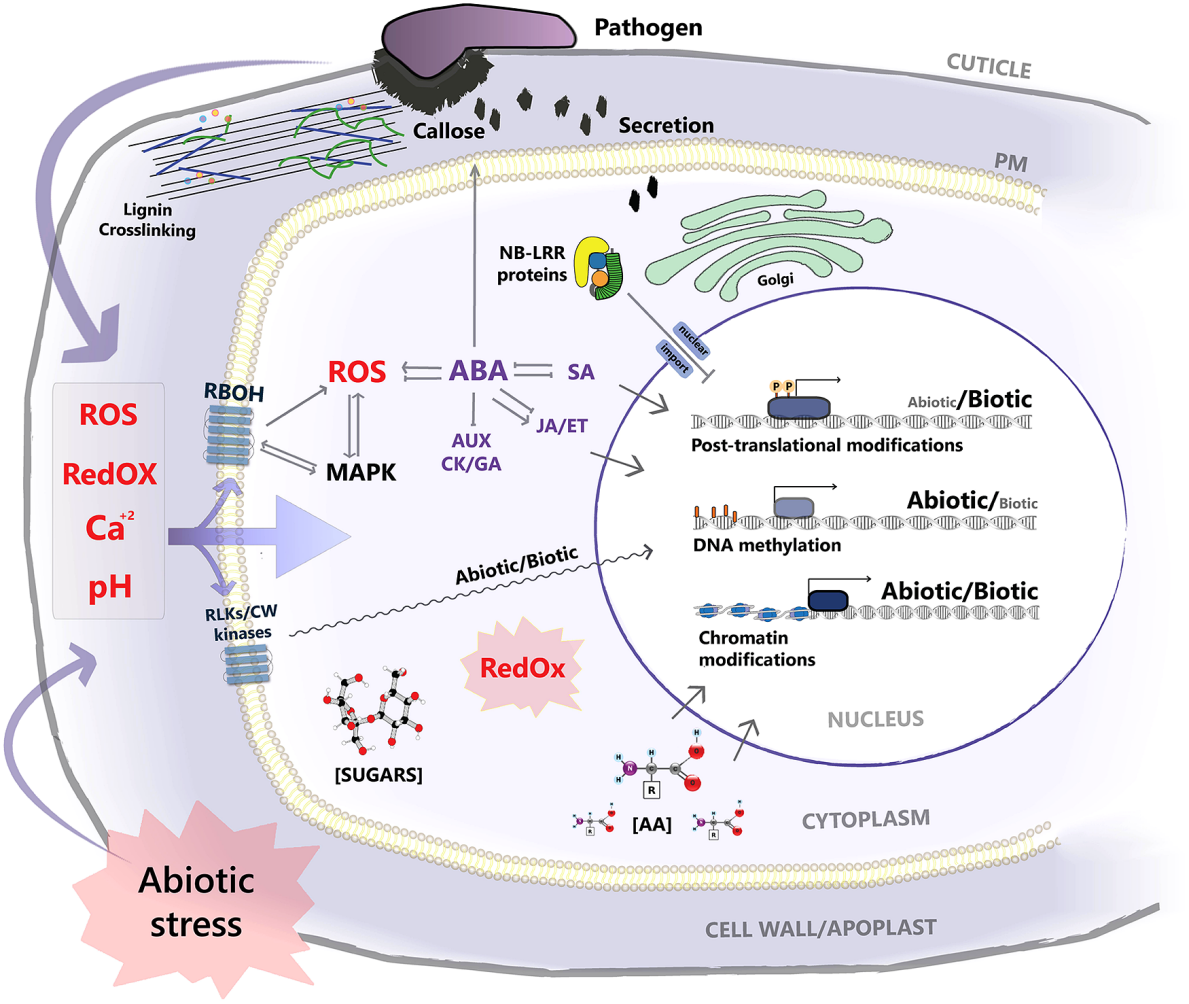
**A scheme for the interaction interface and overlapping signaling pathways of abiotic and biotic stress at the cellular level**. Both stress factors affect the homeostasis of chemical signals at the apoplastic space such as Ca^2+^, ROS, and pH levels. Abiotic stress potentially affects the structure and properties of preformed and inducible physical barriers that function against pathogen penetration. Signaling nodes such as RBOHs and RLKs and other cell wall (CW) kinases localized at the plasma membrane, and MAPKs are shared by both stressors, with downstream signal specificity under stress combination remaining elusive. ABA signaling, central for adaptation to abiotic stress, negatively impinges on defense hormone signaling, while, pathogen dependent, positive interactions are observed for JA signaling. ABA–SA interaction is two sided, as activation of SA signaling by pathogen challenge attenuates ABA responses. ABA positively contributes to pre-invasion defense, enhancing callose deposition. Rewiring of secretory machinery under abiotic stress potentially affects its function in the exocytosis of antimicrobial compounds at the site of infection. Nuclear translocation of R-genes is negatively affected under abiotic stress. Redox state, as well as metabolite concentration such as sugars and amino acids (AA), function as drivers for post-translational modifications, modulating the activity of target proteins/transcription factors. Previously/simultaneously encountered stress effect on chromatin and DNA methylation status, potentially impacts on expression patterns of the recipient genes under stress combination. Transcription factor activation and binding to stress responsive gene promoters is a convergence point regulating the signal output under combinatorial stress with diverse outcomes.

There is evidence that different levels of abiotic stress have a significantly different impact on disease susceptibility (Soliman and Kostandi, 1998; Desprez-Loustau et al., 2006). Salinity stress, in particular, exerts its damaging effect through both osmotic effects and ion toxicity resulting from ion accumulation (mainly Na^+^ and Cl^-^). As NaCl is an antifungal agent (Blomberg and Adler, 1993) it could potentially exert a direct toxic effect on fungal growth after accumulation inside the plants (**Figure 1**). In line with this argument are the many examples of reduction of fungal pathogenicity by metal accumulation (Poschenrieder et al., 2006; Fones et al., 2010), and a similar trend is observed for NaCl accumulation (Soliman and Kostandi, 1998). Therefore salt stress–pathogen interactions may be highly influenced by stress intensity, which affects the degree of accumulation of salt in the plant. The different tolerance strategies of the host against ion toxicity (ion exclusion at the roots and/or ion compartmentalization in the above ground organs inside the vacuoles) can impact on the outcome of plant–pathogen interactions under salt stress. Therefore, it appears that the outcome of the interaction in most occasions is plant, genotype, pathogen, and stress intensity dependent. Moreover abiotic stress, except for potentially dampening or strengthening signaling responses for pathogen defense deployment, could create more or less favorable conditions for pathogen growth by additionally influencing the physiological status of the plant such as water and ion content.

Vice versa, plant responses to abiotic stress can be affected by prior interactions with pathogenic fungi. Pathogen infection has been shown to reduce photosynthesis and water use efficiency (WUE) and induce abnormal stomata opening patterns, and all of these are critical for plant tolerance to abiotic stress (Bilgin et al., 2010; Grimmer et al., 2012). Salicylic acid (SA) signaling, induced after infection with biotrophic fungi, can attenuate abscisic acid (ABA) signaling that is orchestrating plant adaptive responses to abiotic stress (Kim et al., 2011b). Infection by a root pathogen was shown to increase shoot Na^+^ and Cl^-^ content under saline conditions in *Phaseolus vulgaris* (You et al., 2011; **Figure 1**). Finally genetically heightened resistance to pathogens is often accompanied by a fitness cost that may generally affect the plant performance under both abiotic stress and stress-free conditions (Huang et al., 2010; Todesco et al., 2010).

A direct interaction of pathogen virulence factors with stress tolerance components of the plant host was demonstrated for the *Pseudomonas syringae* type III effector HopAM1 that targets HSP70 (Jelenska et al., 2010) involved in heat tolerance and stomata closure under stress (Clement et al., 2011). Overexpression of HopAM1 in *Arabidopsis thaliana* results in increased sensitivity to ABA and salt stress, providing proof of direct manipulation of abiotic stress signaling components (Goel et al., 2008).

Interaction of plants with microorganisms can also be beneficial to abiotic tress tolerance. For instance, infection of plants with RNA viruses improved tolerance to drought (Xu et al., 2008). Infection with the vascular pathogen *Verticillium* spp. increased *Arabidopsis thaliana* drought tolerance due to *de novo* xylem formation, which enhances water flow (Reusche et al., 2012). Symbiosis with fungal endophytes (Marquez et al., 2007) as well as association of plant roots with non-pathogenic rhizobacteria and mycorrhizal fungi increases plant vigor under stress conditions through, among others, interactions with hormonal pathways and the sustainment of water and source-sink relations (Dodd and Perez-Alfocea, 2012). Remarkably, rhizobacteria colonization is also shown to enhance plant resistance to fungal pathogens and insects, via systemic signaling that triggers immunity (induced systemic resistance, ISR; Berendsen et al., 2012).

Further evidence for abiotic and biotic stress resistance crosstalk comes from studies of the effects of exogenous application of chemicals that sensitize plant defense responses, a phenomenon called priming (Goellner and Conrath, 2008). For example, application in *Arabidopsis thaliana* of β-aminobutyric acid (β-ABA), a non-protein amino acid, results in enhanced resistance to a wide range of stresses including heat, drought, and salinity stress, as well as enhanced resistance to biotrophic as well as necrotrophic fungi (Ton et al., 2005). Exogenous application of SA renders many crop plants more tolerant to an extensive array of abiotic stresses (Horváth et al., 2007), and similar observations have also been reported after treatment with jasmonates (Walia et al., 2007).

### EVIDENCE FOR CROSSTALK FROM WHOLE GENOME EXPRESSION ANALYSES

Evidence for regulatory crosstalk between abiotic and biotic stress response at the molecular level comes mostly from observations of expression patterns of genes under independent imposition of the single stress conditions. In *Arabidopsis thaliana*, a significant number of genes up-regulated by salinity stress are also induced in response to biotic stresses (Ma et al., 2006). Whole genome expression meta-analysis experiments under different abiotic and biotic stress treatments revealed a significant number of genes that are commonly regulated under abiotic and biotic stress conditions (Ma and Bohnert, 2007; Shaik and Ramakrishna, 2013, 2014). Functional categories enriched in the 197 commonly regulated genes identified by (Ma and Bohnert, 2007) include response to ABA, SA, jasmonic acid (JA), and ethylene (ET), major stress hormones controlling adaptation to abiotic and biotic stress. Several members of signaling pathways involving mitogen-activated protein kinase (MAPK), Ca^2+^, ROS, phospholipids, mitochondrial functions, vesicle trafficking, and apoptosis were induced under biotic as well as abiotic stresses (Ma and Bohnert, 2007). Transcription factors (TFs) appear to be major orchestrators of stress crosstalk with members of WRKY, MYB, ERF, NAC, and HSF displaying similar induction patterns across stress treatments (Ma and Bohnert, 2007; Shaik and Ramakrishna, 2013). On the other hand, another study using co-expression data to identify *cis*-regulatory elements (CREs) of stress responses identified distinct CREs for the response to abiotic and biotic stressors (Zou et al., 2011). In addition, a number of CREs identified for both types of stress appear to oppositely regulate the expression of downstream genes in response to abiotic or biotic stress.

A different approach, yeast two-hybrid assays targeting major regulators of rice abiotic and biotic stress response, identified proteins that are present in multiple interactomes (Seo et al., 2011; Sharma et al., 2013). These include OsMPK5, the wall-associated kinase 25 (WAK25), sucrose non-fermenting-1-related protein kinase-1 (SnRK1), and WRKY family TFs.

Recently, examination of the transcriptional response of different *Arabidopsis thaliana* accessions to combinations of abiotic and biotic stressors revealed that across the treatments on average 60% of expression changes under combinatorial stress could not be predicted by the changes in response to the individual stresses (Rasmussen et al., 2013). The functional categories enriched in the affected genes were similar to those discovered after transcriptome meta-analyses of individual stressors, i.e., stress hormone responses, ROS, and MAPK signaling and regulation of hypersensitivity response. The response of many of these transcripts was canceled or prioritized under stress combination in comparison with the individual stress pointing to potential antagonistic interactions with detrimental effects on plant adaptation under combinatorial stress. In a similar study, the increased susceptibility to a virus after simultaneous application of drought and heat stress was accompanied by down regulation of pathogenesis-related (PR) genes and R-genes, which were otherwise induced under single viral stress (Prasch and Sonnewald, 2013). This indicates a direct negative effect of abiotic stress on major defense executors, that adds up to the antagonistic regulation observed in other signaling pathways. These studies clearly emphasize that even though regulatory pathways overlap between stresses, combinatorial stress needs to be treated and studied as a unique condition. Further functional characterization of individual gene members playing key roles in these pathways is required to extract meaningful conclusions.

## ABIOTIC–BIOTIC STRESS INTERACTION INTERFACE

**FIGURE 2:**
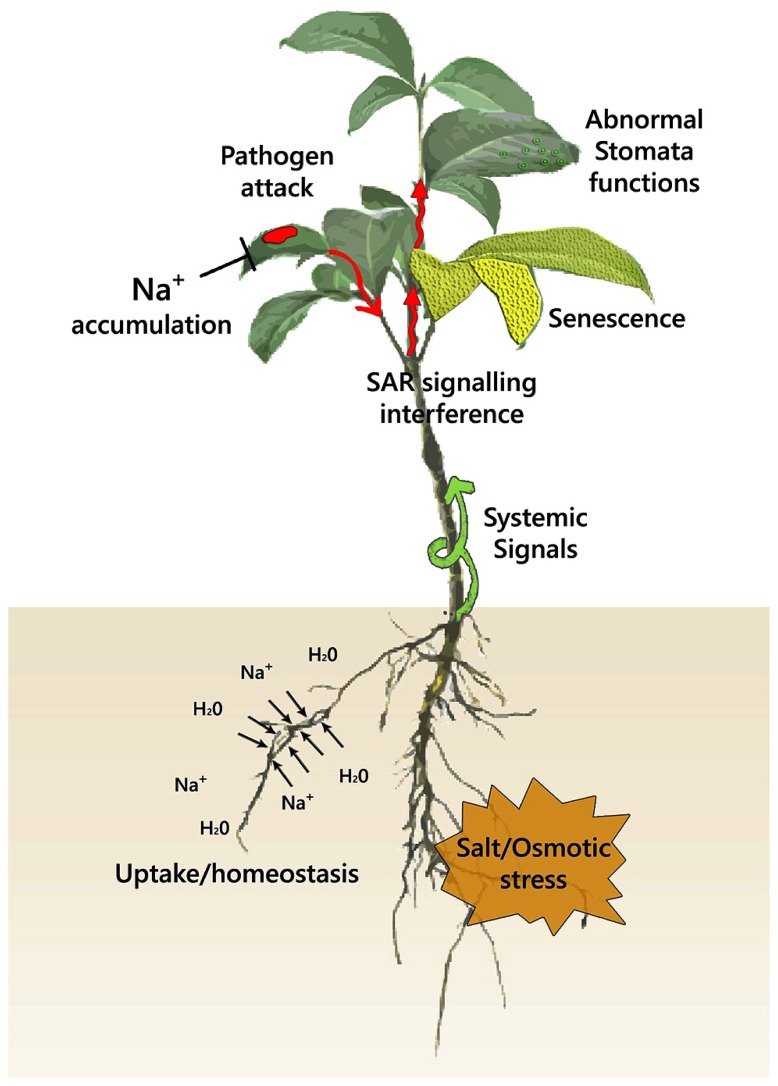
**A scheme for the effects of abiotic and biotic stress at the plant level**. A combination of abiotic stress with pathogen infection potentially derails hormone and systemic ROS homeostasis. Pathogen infection has been shown to impair stomata closure under non-stress conditions, with the dynamics of this interaction under abiotic stress being unknown. Senescence is a common component of both abiotic and biotic stress that can potentially be amplified under combinatorial stress. Systemic ROS signals generated after pathogen encounter may alter water relation and salt uptake through their effects in root hydraulic conductance and ion transport. Abiotic stress through ABA signaling negatively affects signals that trigger systemic acquired resistance, enhancing pathogen spread from the initial site of infection. Ion accumulation (Na^+^, Cl^-^) under salt stress can have a direct toxic effect on pathogen growth.

As mentioned above, abiotic and biotic stress interactions can occur at multiple levels, depending on the type of the stress (osmotic, ionic), the lifestyle, and infection strategy of the pathogen (biotroph/necrotroph, infection by direct penetration/through stomata, etc.) as well as the pathogenesis stage. We will summarize molecular components that according to evidence mentioned above participate in stress crosstalk. We will follow the pathogenesis timeline highlighting first extracellular interactions taking place at the epidermis and the apoplast during the initial stages of pathogenesis and moving on to the interactions in the intracellular environment during pathogen colonization (**Figure 2**). As information under combined stress is limited, and a detailed coverage of all potential interactions is not possible, our intention is to provide leads for future research that will aid to further dissect plant adaptive responses and tolerance under combined abiotic and biotic stress.

### EXTRACELLULAR INTERFACE

#### Cuticular layer

The cuticle and cell wall constitute the first layers of defense against microbial attackers. They not only serve as physical barriers against pathogen penetration, but also as sensitive sensors for the timely activation of the intracellular and systemic defense responses. *Arabidopsis thaliana* mutants in long-chain acyl-CoA synthetase 2 (LACS2), a gene that is involved in cuticle biosynthesis, exhibit increased permeability of the cuticular layer which leads to increased resistance to *Botrytis cinerea* (Bessire et al., 2007). Interestingly, ABA deficiency causes similar cuticular defects and heightened resistance to *B. cinerea* through faster induction of defense responses and H_2_O_2_ production both in *Arabidopsis* and tomato, indicating a link between abiotic stress signaling, cuticle structure and defense responses (Curvers et al., 2010). In the study by Xiao et al. (2004), however, *lacs2 Arabidopsis* mutants show no alteration in the resistance against the necrotroph *Alternaria brassicicola* and biotrophs, and even increased susceptibility against *P. syringae*. The latter observation points to a positive contribution of a thicker cuticle to resistance against *P. syringae*, indicating that the effects may be pathogen-specific (Tang et al., 2007). The well-documented increase in cuticular thickness under conditions of water deficiency (Kosma et al., 2009) may thus result in alteration in the deployment of the pathogen defense response. The cuticle does appear to be a sensor of the osmotic status and to be essential for the up-regulation of ABA biosynthesis genes under osmotic stress (Wang et al., 2011) through a yet not clearly defined mechanism; cuticle disruption by pathogens may therefore affect osmotic stress acclimation.

#### Cell wall-apoplastic space

Cell walls similarly appear to be an integrated signaling component for the defense against pathogens. Changes in pectin properties and composition in the *Arabidopsis* powdery mildew-resistant (*pmr*) mutants *pmr5* and *pmr6* resulted in a SA, JA, and ET independent increase in resistance to powdery mildew species (Vogel et al., 2004). Cellulose deficiency caused either by non-functional cellulose synthase genes or by chemical treatment enhances the synthesis of the defense hormones SA, JA, and ET and signaling and results in increased resistance to pathogens (Hématy et al., 2009). Intriguingly, these responses were attenuated when plants were grown under high osmotic pressure which reduced the turgor pressure (Hamann et al., 2009), suggesting that the defense response may be initiated by sensing the increased turgor pressure as a result of cell wall weakening. Osmotic stress, which is a common component of many abiotic stresses, may therefore interfere with the ability of plants to sense damage to the cell wall, due to already reduced turgor, resulting in inadequate activation of defense mechanisms.

The above-mentioned alterations in plant pathogen interactions in cell wall component biosynthesis mutants may be the consequence of the erroneous activation of integral receptor proteins such as RLKs and RLPs (receptor-like kinases and receptor-like proteins, respectively) which survey the cell wall integrity and bind to MAMPs and DAMPs (microbial- and damage-associated molecular patterns, respectively). Upon activation these transmembrane proteins (e.g., the RLK family WAK), send signals for the elicitation of downstream defense responses. Changes of cell wall structure and adherence to the plasma membrane upon exposure to abiotic stresses may affect their functional integrity. This is emphasized by the observation that NDR1, an essential component of disease resistance mediated by CC-NB-LRR genes (McHale et al., 2006), is functioning in cell wall-plasma membrane adhesion. Down-regulation of NDR1 resulted in alterations in the cell wall-plasma membrane interaction and compromised resistance to virulent *P. syringae* (Knepper et al., 2011). Abiotic stress may also affect the abundance of cell wall receptors by influencing their transcript levels. THE1 is a member of the CrRLK1L RLK family that is involved in cell wall damage sensing and subsequent control of the downstream accumulation of ROS, and its expression is down-regulated under abiotic stress but up-regulated after pathogen challenge (Lindner et al., 2012), while similar expression patterns are observed for the WAK gene family (Shaik and Ramakrishna, 2013).

Pathogen recognition activates a battery of defense responses that target the apoplastic space. These include local cell wall enforcement, secretion of antifungal compounds at the site of intended penetration and up-regulation of enzymes with fungal cell wall degrading activities (Van Loon et al., 2006). These events are characterized and regulated by signature changes in pH, ROS homeostasis, and the redox state. Simultaneous exposure to abiotic stress can potentially impinge on the generation and decoding of these signatures, affecting subsequent responses. For example, apoplastic pH is transiently decreased following fungal infection (Felle et al., 2004), while an increase in pH is observed under salt stress (Geilfus and Muhling, 2011). Moreover the downregulation of cell wall peroxidases under abiotic stress (Shaik and Ramakrishna, 2014) can potentially dampen the production of ROS signatures that trigger defense responses (Daudi et al., 2012). Physical barriers enforcement after pathogen encounter through crosslinking of lignin monomers by ROS, which are produced by apoplastic peroxidases, NADPH oxidases and germin-like proteins, prevent pathogen penetration. Lignin content was found to be reduced under mild drought conditions to facilitate the maintenance of growth under conditions of decreased turgor pressure (Vincent et al., 2005), but severe stress resulted in increased lignin content (Lee et al., 2007). These findings may provide insight on the mechanisms leading to differential responses under combined stress across different abiotic stress intensities.

#### Vesicular trafficking and callose deposition

Another form of inducible defense response at the site of penetration is the formation of papillae that contain callose, antimicrobial secondary metabolites such as phenolic compounds, and ROS. Antimicrobial compounds are accumulating through vesicles originating from cellular compartments, such as the Golgi apparatus, which become polarized toward the site of infection (Underwood and Somerville, 2008). The significance of vesicle-mediated secretion in plant immunity has been demonstrated by the discovery of mutants defective in exocytosis of vesicles (with mutations in SNARE complex proteins *Hv*ROR2 and *At*PEN1), which display diminished penetration resistance to powdery mildew pathogens (Collins et al., 2003). Vesicular trafficking appears to be rewired in an opposite way under salt stress, as vesicles containing Na^+^ are fused with the central vacuole to maximize compartmentalization of Na^+^ (Hamaji et al., 2009). Interestingly, knockout of different SNARE proteins resulted in increased salt tolerance (Hamaji et al., 2009), indicating possible antagonistic interactions of salt stress and pathogen infection at the level of vesicle trafficking, although further comprehensive experiments are required to substantiate this hypothesis.

Callose is a β-1,3-glucan polymer that is deposited at the sites of attempted fungal penetration in the form of papillae. It is an important inducible defense mechanism, with enhanced deposition being observed after exogenous application of priming chemicals like β-ABA. A mutant screen for plants defective in β-ABA-induced priming identified among others mutants in the ABA biosynthesis gene *zeaxanthin epoxidase* (ABA1; Ton et al., 2005). These mutants failed to exhibit both β-ABA-induced callose deposition against *H. parasitica* and increased tolerance to salt stress, thereby providing a link between the induction of abiotic and biotic stress responses by β-ABA. In accordance with these observations the callose-mediated increased resistance of the *ocp3 Arabidopsis* mutant to necrotrophic pathogens requires ABA (Garcia-Andrade et al., 2011). Moreover *ocp3* mutants accumulate higher levels of ABA, and are more drought tolerant (Ramírez et al., 2009). Therefore ocp3, a homeodomain TF, appears to be a convergence point for ABA and callose regulation that can be manipulated to enhance resistance under combinatorial stress.

Callose accumulation appears to be a point of convergence of abiotic and biotic signaling as variability in environmental conditions, which affect the redox state of the plant, such as light intensity, have a significant impact on the magnitude of callose deposition after pathogen elicitation (Luna et al., 2011). As callose deposition is a major component of the pre-invasion defense of plants (Ellinger et al., 2013), detailed characterization of the regulation of callose accumulation under simultaneous abiotic stress may be invaluable in building combined stress tolerance in crops.

### INTRACELLULAR SIGNALING INTERACTIONS

#### Interconnections between Ca^2+^ and ROS signaling

Changes in calcium fluxes and production of ROS are among the earliest plant responses to abiotic stress and pathogen challenge. The decoding of both signals relies on “signature” spatiotemporal patterns and oscillations specific to the stress encountered (Dodd et al., 2010; Mittler et al., 2011). Moreover, both components are highly interconnected: Ca^2+^ signaling components such as calmodulins (CaMs) and calcium-dependent protein kinases (CDPKs) regulate ROS production by NADPH-oxidases (Takahashi et al., 2011). ROS vice versa affect Ca^2+^ signaling through regulation of Ca^2+^ permeable channels (Demidchik et al., 2007). It is plausible that there are either unique signatures for combinations of stresses, or that there is interference between the abovementioned signals that potentially dampens or strengthens the downstream responses.

Whole genome expression analyses coupled with promoter motif identification provided further evidence that Ca^2+^ orchestrates the early responses to both biotic and abiotic stress as the overrepresented motif “CGCGTT” identified in the promoters of the commonly regulated genes, contains the core “CGCG” Ca^2+^ responsive *cis*-element (Walley et al., 2007). The investigation of mutants defective in the induction of a hypersensitive response after pathogen infection has led to the identification of genes encoding for cyclic nucleotide gated channels (CNGCs) which are non-selective cation transporters (Clough et al., 2000). members of which are also involved in salt and heat stress tolerance through regulation of Ca^2+^ fluxes (Guo et al., 2010; Finka et al., 2012). Furthermore, Ca^2+^ downstream signaling components have been shown to mediate responses to both abiotic and biotic stress stimuli. The CAMTA3 TF is important for cold acclimation of *Arabidopsis* by stimulating the expression of CBF1, CBF2, and ZAT12 that are also involved in adaptation to dehydration and oxidative stress (Doherty et al., 2009). Moreover, *C*AMTA3 negatively regulates SA accumulation and plant defenses through CaM binding (Du et al., 2009). Other proteins interacting with CaM include TF families like NAM, WRKY, and MYB (Popescu et al., 2007) many members of which are involved in abiotic and biotic stress crosstalk.

*CDPKs* have a unique feature to both bind calcium and functionally decode the message by target protein phosphorylation. They appear to represent a central node in the regulation of abiotic and biotic stress responses (Schulz et al., 2013). For example, *Arabidopsis* CPK4 and CPK11 positively regulate ABA responses and their down-regulation renders plants salt-sensitive (Zhu et al., 2007), and are important for the oxidative burst and defense responses (Boudsocq et al., 2010). In addition, CDPKs regulate ROS production through phosphorylation-mediated regulation of RBOH activity (Dubiella et al., 2013). *St*CDPK4- and *St*CDPK5-mediated phosphorylation increases the activity of StRBOHs and the increased ROS production results in a stronger hypersensitivity response after pathogen challenge, favoring resistance against biotrophic pathogens but compromising resistance against necrotrophic fungi (Kobayashi et al., 2012). Recently, the CDPK OsCPK12 was shown to increase salt stress tolerance and decrease blast disease resistance in rice through reduced ROS production as a result of down-regulation of RBOH expression, enhanced expression of antioxidant genes such as APX (ascorbate peroxidase), and increased sensitivity to ABA (Asano et al., 2012).

Dissecting the spatiotemporal and molecular specificity of Ca^2+^ and ROS signaling components is crucial for determining their precise functions in stress responses (Baxter et al., 2014), as is elegantly demonstrated by the identification of different Ca^2+^ binding affinities regulating the activation of two soybean CaMs (Gifford et al., 2013).

#### Signal relay by MAPKs

Mitogen-activated protein kinases are centrally positioned in Ca^2+^–ROS crosstalk and regulation as well as in the signal output after stress exposure. MAPK signaling cascades are relayed through MAPK kinase kinases (MAP3Ks) and MAPK kinases (MAP2Ks). Hydrogen peroxide (H_2_O_2_) has been shown to mediate activation of the three major and well-studied *Arabidopsis* MAPKs, MAPK3, 4, and 6, through MAP3Ks and other kinases (Rentel et al., 2004; Teige et al., 2004). These MAPKs appear to have an overlapping function in signal transduction upon abiotic stress and pathogen challenge. Activation of *Arabidopsis* MAPK3 and MAPK6 as well as their homologs in tobacco WIPK and SIPK (Segonzac et al., 2011) after PAMP recognition is essential for fungal and bacterial resistance (Asai et al., 2002). The importance of MAPK3 and MAPK6 in plant immune responses is highlighted by the discovery that the *P. syringae* effector HopAI1 directly interacts and inactivates both, promoting virulence (Zhang et al., 2007). Additionally MAPK6 is directly involved in regulating ET biosynthesis in *Arabidopsis* by activation through phosphorylation of ACS2 and ACS6, which results in an increase in ET biosynthesis (Liu and Zhang, 2004). MAPK4 acts as a negative regulator of defense responses and SA accumulation by phosphorylating MEKK2, a MAP3K protein (Kong et al., 2012).

On the other hand, down-regulation of MAPK3 resulted in altered stomata opening patterns in response to ABA and H_2_O_2_ in *Arabidopsis* (Gudesblat et al., 2007). Moreover, the ABA-induced expression of AtCAT1, which is involved in H_2_O_2_ homeostasis, is controlled by an AtMKK1–AtMAPK6 signaling cascade (Xing et al., 2008). Constitutive activation of AtMAPK4 and AtMAPK6 rendered plants more tolerant to cold and salt stress (Teige et al., 2004) and CAT2 and tAPX, which are involved in H_2_O_2_ regulation, appear to be regulated by AtMAPK4 (Pitzschke et al., 2009). In rice, OsMAPK5 appears to be a convergence point of abiotic and biotic stress responses, as its silencing results in sensitized defense responses and resistance to fungal and bacterial pathogens at the expense of salt and drought tolerance (Xiong and Yang, 2003).

These examples emphasize the complexity of MAPK-mediated defense signaling with diverse and sometimes overlapping functions of different members of the signaling pathway. Downstream targets of MAPK6 overlapped 60% with MAPK3 targets, while a 50% overlap was observed between MAPK3 and MAPK4 targets (Popescu et al., 2009). Probably, the one-dimensional overlap can be resolved by multidimensional regulation, such as different spatiotemporal transcription and protein subcellular localization, activation thresholds, feedback loops with phosphatases and scaffolding (Tena et al., 2011; Samajova et al., 2013). Many of the above-mentioned components appear to be an integral part of broad stress tolerance priming by exogenous application of chemicals (Beckers et al., 2009; Xia et al., 2009), and the detailed study of MAPK activation, localization, and substrate affinity under these conditions can increase our understanding of plant responses under stress combinations.

#### Hormone signaling

Plant hormones are central to the integration of environmental stimuli in the coordination of growth under optimal and stress conditions, including the regulation of defense responses after pathogen attack. Plant hormones do not act independently, and extensive synergistic or antagonistic interaction between hormonal pathways is observed in development and stress responses after exogenous application, or through mutant analysis (Wolters and Jurgens, 2009). Transcriptomic studies have aided in unveiling these interactions (Nemhauser et al., 2006), and it was recently shown that hormonal pathways can be directly connected with each other by protein–protein interactions between their signaling components (Hou et al., 2010; Zhu et al., 2011).

Abscisic acid is the major hormone that positively contributes to adaptation to osmotic stress, a major component of several abiotic stresses. Its involvement in the regulation of defense responses has been a topic of recent comprehensive reviews (Asselbergh et al., 2008; Ton et al., 2009). The consensus is that ABA negatively regulates defense responses against both biotrophic and necrotrophic pathogens through negative interactions with SA and JA/ET biosynthesis and signaling; ABA biosynthesis mutations show sensitization of these signaling pathways after pathogen challenge (Achuo et al., 2006; De Torres Zabala et al., 2009; Sanchez-Vallet et al., 2012). Comprehensive analyses of ABA-deficient mutants revealed further pleiotropic alterations that may be part of ABA-defense crosstalk such as reduced cuticle thickness and sensitized H_2_O_2_ production in response to *B. cinerea* in tomato (Asselbergh et al., 2007) and altered cell wall composition in *Arabidopsis* (Sanchez-Vallet et al., 2012). Moreover ABA compromised a chemically induced systemic acquired resistance (SAR) through suppression of SA biosynthesis in *Arabidopsis*, while genetically enhanced ABA catabolism reversed this effect (Yasuda et al., 2008).

Nevertheless, ABA signaling can positively contribute to pre-invasive defense responses and to early defense signaling against certain necrotrophic pathogens (Adie et al., 2007). ABA positively contributes to resistance against pathogens that infect through stomata, such as *P. syringae* (Melotto et al., 2006), as well as to other pre-invasion defense mechanisms such as callose deposition (Ton and Mauch-Mani, 2004; Adie et al., 2007; Garcia-Andrade et al., 2011).

Identification of downstream regulatory nodes that channel interactions between hormonal pathways is of great importance in fine-tuning resistance to both abiotic and biotic stress. Besides TFs, which will be discussed in a following section, other regulators of the transcriptional machinery have been uncovered to function in stress crosstalk. RNA chaperones such as RNA helicases are shown to regulate transcription in response to various stressors (Li et al., 2008; Mazzucotelli et al., 2008). MED25, a subunit of the mediator complex which is a component of the transcriptional machinery, is involved in the antagonistic crosstalk between ABA and JA (Chen et al., 2012). In a recent report, the *Arabidopsis* pathogenesis-related protein 2 (PR2), which encodes β-1,3-glucanase involved in callose degradation, was shown to be down regulated in response to ABA, partly elucidating ABA-mediated capacitation of callose deposition. The *ahg2-1* mutant in *Arabidopsis* accumulates both ABA and SA and has increased expression of defense related genes, which is an indication that ABA and SA do not always act antagonistically. Transcriptome analysis of the *ahg2-1* mutant revealed complex interactions between ABA and SA signaling involving altered mitochondrial and RNA metabolism (Nishimura et al., 2009), highlighting multilevel connections between the two signaling pathways that add to the complexity and hinder straightforward conclusions.

Recent research has highlighted the direct involvement of the growth hormones gibberellin, cytokinin, auxin, and brassinosteroid in responses to adverse growth conditions and pathogen attack (Robert-Seilaniantz et al., 2011). For example, GA signaling directly regulates JA signaling, mediated through direct binding of the GA repressor protein DELLA to JAZ proteins and relieving JA signaling repression (Hou et al., 2010). DELLA proteins appear to be central nodes in abiotic and biotic stress cross-talk. ABA and ET signaling promote DELLA stabilization which positively affects ROS detoxification (beneficial for acclimation to abiotic stress) through higher expression of ROS detoxification genes (Achard et al., 2008). DELLAs also sensitize JA signaling (through binding of DELLAs to JAZ) at the expense of SA signaling, enhancing resistance to necrotrophic pathogens (Navarro et al., 2008). This may provide an explanation for the often-observed positive correlation between resistance to abiotic stress and resistance to necrotrophs (Navarro et al., 2008; Abuqamar et al., 2009; Ramírez et al., 2009).

Cytokinins were shown to positively regulate defense responses to biotrophic pathogens (Argueso et al., 2012) via SA accumulation, and increased defense gene expression through interaction of the cytokinin response regulator ARR2 with TGA3, a TF central for defense gene activation (Choi et al., 2010). This suggests that the increased cytokinin catabolism observed under abiotic stress-induced senescence may potentially contribute to further down-regulation of SA responses and increased susceptibility to biotrophic pathogens.

The roles of auxin and brassinosteroids in stress responses and their potential participation in stress crosstalk remains elusive. Auxin signaling shows antagonistic crosstalk with SA (Wang et al., 2007), although auxin contributes to reduced senescence (Kim et al., 2011a) which may be of great importance under exposure to a stress combination. Brassinosteroid (BR) signaling positively affects abiotic stress tolerance, as is evident by both BR exogenous application (Divi et al., 2010) and genetic de-repression of the BR signaling pathway (Koh et al., 2007). BR signaling probably interacts synergistically with ABA signaling and stimulates ROS detoxification (Divi et al., 2010). BR’s involvement in defense signaling is rather complicated. In tobacco and rice exogenous application of BRs appeared to clearly enhance resistance to a wide range of pathogens (Nakashita et al., 2003). Similar results were obtained in cucumber, which showed heightened resistance to *Fusarium oxysporum* as a result of activated production of H_2_O_2_ by NADPH oxidase and expression of defense related genes (Li et al., 2013). On the contrary BRs appear to be negatively regulating resistance to the root-infecting oomycete *Pythium graminicola* by antagonizing SA and GA related defense responses (De Vleesschauwer et al., 2012). BR signaling shares LRR–RLK and BAK1 proteins with PAMP immune signaling (Chinchilla et al., 2009). Contradictory effects of BR signaling on immune responses have been recently reported in *Arabidopsis* (Albrecht et al., 2012; Belkhadir et al., 2012; Lin et al., 2013), which require further study.

It is clear that hormonal crosstalk is extensive and occurs in multiple combinations. Further understanding of plant responses under combined stress exposure is required to dissect the multilevel responses under these conditions. As an example of the underlying complexity, both drought stress and exogenous ABA application result in an increased endogenous ABA content in tomato, but they differentially affect resistance to powdery mildew and *Botrytis*, with drought enhancing and ABA application compromising resistance (Achuo et al., 2006). Notably the ABA-deficient tomato mutant *sitiens* exhibited increased resistance similar to the effect of drought (Achuo et al., 2006). The complexity of interactions under abiotic stress is further emphasized by transcriptome analyses under abiotic stress in which up-regulation of a significant number of JA/ET-responsive genes and accumulation of their transcripts was observed (Walia et al., 2007; Huang et al., 2008). Besides the effects of direct hormonal interactions on abiotic and biotic stress tolerance mechanisms additional indirect interactions should be considered, such as the alteration of developmental programs and the regulation of senescence which may be critical for evolutionary species fitness and yield performance in crop plants (Wu et al., 2012a).

#### Cellular redox state

The cellular redox state is the sum of reducing and oxidizing redox-active molecules (Potters et al., 2010) and it acts both as a sensor of environmental perturbations (as most of them impose oxidative stress) and as a buffer against these perturbations to maintain cellular homeostasis. It acts as a central integrator of ROS, energy and metabolic regulation under stress as well as optimal conditions. Its major constituents are ascorbate, glutathione (GSH), NADP(H), small proteins acting as antioxidants like thioredoxin and glutaredoxins as well as many diverse metabolites such phenolics, amino acids, carotenoids, and tocopherols. The cellular redox state is dependent on both their accumulation and their reduction-oxidation state (Potters et al., 2010). Genetic manipulation of redox homeostasis results in altered hormone homeostasis and responses to pathogens and abiotic stresses (Mhamdi et al., 2010), exemplifying its significance. As abiotic and biotic stress commonly impinge on the redox status (albeit not in a similar manner; Foyer and Noctor, 2005), redox homeostasis is potentially a central orchestrator of the phenotypic response to stress combinations. Redox perturbations after imposition of a stress factor may affect responses to subsequent challenges by additional stressors, thereby shaping the response to combined stresses. For example, a transient increase in GSH content drives the antagonistic crosstalk between SA and JA signaling (Koornneef et al., 2008) and GSH oxidation appears to drive the induction of both SA and JA pathways (Mhamdi et al., 2013).

Plant hormone signaling can directly perturb the redox status by modifying the expression and activities of antioxidant enzymes. ABA induces the expression of catalase, activating also at the same time the production of the ROS hydrogen peroxide through AtMAPK6 signaling (Xing et al., 2008). SA inhibits the function of catalase and cytosolic ascorbate peroxidase (Corina Vlot et al., 2009) and several glutathione transferases (Tian et al., 2012).

Programed cell death (PCD) is a plant response to developmental and environmental stimuli (e.g., in senescence) and pathogen defense (in the form of HR) that is initiated and regulated by redox changes, like an increased oxidation ratio of GSH and ascorbate (De Pinto et al., 2012). APX appears to be central in the redox regulation leading to PCD. Decreased activity of APX isoforms was observed in heat-induced PCD (Locato et al., 2009), and overexpression or down-regulation in *Arabidopsis* of a thAPX increased or decreased, respectively, sensitivity to NO-induced cell death (Tarantino et al., 2005). APX isoforms are also commonly up-regulated under abiotic stress (Miller et al., 2008). Considering the important role of APX in the drought–heat stress interaction (Koussevitzky et al., 2008) it is of great interest to explore APX enzyme regulation under combinatorial stress.

Redox status changes can directly impact protein function through post-translational modifications. One pronounced example of post-translational modifications controlling protein activity and localization is the interplay of *S*-nitrosylation and thioredoxin-mediated reduction in the control of the oligomeric and monomeric state of NPR1 (Tada et al., 2008), a master regulator of SA-mediated defense responses and recently proposed as a SA receptor (Wu et al., 2012b). The function of many more proteins appears to be regulated by *S*-nitrosylation, among them AtRBOHD as mentioned above (Yun et al., 2011), SA binding protein 3 (SABP3), methionine adenosyltransferase 1, the metabolic enzymes glyceraldehyde 3-phosphate dehydrogenase (GAPDH) and glycine decarboxylase (GDC), as well as metacaspase 9 (Astier et al., 2011). Identification of the dynamics of post-translational modifications on these and newly identified proteins under various stress combinations will shed light on their significance for plant adaptation responses to these conditions.

NO was recently found to exhibit biphasic control over cell death triggered by pathogens and pro-oxidants in *Arabidopsis*. In initial stages *S*-nitrosothiol (SNO) accumulation results in enhanced and accelerated cell death (Yun et al., 2011). However, constitutively high SNO levels decreased cell death through *S*-nitrosylation-mediated reduction in AtRBOHD activity (Chen et al., 2009; Yun et al., 2011). This differential regulation might have implications in conditions of combined abiotic and biotic stress as both result in increased NO levels. At a certain plateau concentration of NO, signaling components may be desensitized or inversely regulated, as exemplified by AtRBOHD, with detrimental effects on stress acclimation.

Redox changes and post-translational modification appear to be integral in priming for stress tolerance after exogenous application of chemicals (Tanou et al., 2009). This provides a potential explanation of the mechanism of action of diverse chemicals in plant defense sensitization. H_2_O_2_ and NO priming for salt tolerance in citrus moderately increased the abundance of oxidized and *S*-nitrosylated proteins, which then remained relatively similar after the application of stress. Non-treated plants were more stress sensitive and exhibited increased protein carbonylation and oxidation (Tanou et al., 2012). As both compounds provide increased tolerance to both abiotic and biotic stress, further characterization including the timing and magnitude of these post-translational modifications under different stress treatments and under stress combination may help to better understand the redox changes leading to stress cross-tolerance.

#### Metabolite homeostasis and signaling

Metabolites are the end products of gene expression and protein activities and therefore are the penultimate regulatory component for the phenotypic expression under stress conditions. As metabolites can have multiple functions such as being energy carriers, structural molecules and redox regulators or exerting direct antimicrobial activity against pathogens, uncovering their regulation and homeostasis under combined stress is of great significance.

Adaptation to both abiotic and biotic stress impinges significantly on primary metabolism homeostasis. Synthesis of antimicrobial metabolites and defense proteins is energy demanding (Bolton, 2009), while abiotic stress potentially leads to energy deprivation as photosynthesis is reduced under abiotic stress (De Block et al., 2005). As a result, it is fair to assume that under stress combinations these strong antagonistic effects will result in disturbed energy balance. However, recent results challenge the carbohydrate deprivation notion under mild dehydration stress (Hummel et al., 2010) and further experimental data under combined stress are required for firm conclusions. More evidence that sugar homeostasis and signaling drives defense responses are demonstrated by the down regulation of cell wall invertases. This results in dampening of defense responses and increased susceptibility to pathogens as a result of decreased availability of carbohydrates to fuel the defense responses at the site of infection (Essmann et al., 2008). Cell wall invertases appear to be down regulated under abiotic stress (Wingler and Roitsch, 2008) and as the regulation of their activity is a convergence point of hormonal and sugar signals for stress tolerance and senescence progression (Wingler and Roitsch, 2008), fine tuning of their expression might be a focal point in enhancing combined stress tolerance. The metabolic status of the host is also crucial for pathogen growth as it appears that pathogens manipulate different aspects of plant metabolism to achieve optimal conditions for their growth (Chen et al., 2010a).

The significance of amino acid homeostasis for the induction and regulation of defense responses was recently highlighted (Zeier, 2013). Amino acids may function as precursors in hormone biosynthesis and affect the redox state through their chemical properties or as precursors of redox regulators such as GSH. Amino acid abundance can impact hormone signaling through conjugation-mediated regulation of hormone activity (Woldemariam et al., 2012). Amino acid concentration appears to be significantly perturbed by abiotic stress as is revealed by metabolomics studies (Obata and Fernie, 2012). On the other hand a direct link between amino acid abundance and activation of SA-induced defense responses was recently demonstrated with heat-shock factor HsfB1, the translation of which is initiated under conditions of phenylalanine starvation (Pajerowska-Mukhtar et al., 2012). Phenylalanine appears to be accumulated under abiotic stress conditions (Urano et al., 2009; Widodo et al., 2009) and its potential as a molecular switch between abiotic and biotic stress responses should be explored.

Metabolic alterations under abiotic stress include the accumulation of compounds such as the raffinose family oligosaccharides raffinose and galactinol and the amino acid proline. These exhibit osmoprotective and antioxidant functions and have been positively correlated with abiotic stress tolerance (Korn et al., 2010). Galactinol overproduction was recently associated with increased resistance to necrotrophic pathogens (Mi et al., 2008). Moreover, proline metabolic regulation at the site of pathogen infection is important for both HR deployment and containment, probably through modulation of ROS levels as shown by expression and functional studies of proline dehydrogenase (Senthil-Kumar and Mysore, 2012). Myo-inositol metabolic regulation appears to be a convergence point for abiotic and biotic stress responses. Myo-inositol is accumulating under most abiotic stress conditions and is positively contributing to tolerance as a compatible solute (Tan et al., 2013). A negative relationship between myo-inositol accumulation and pathogen resistance and PCD initiation was found in *Arabidopsis*, with a positive correlation between myo-inositol depletion and increased SA production and cell death (Chaouch and Noctor, 2010).

Analysis of mutants that exhibit qualitative and quantitative alterations in the accumulation of fatty acid metabolites demonstrated that fatty acids are not only structural components of the cellular membranes, but they also exert a multitude of signaling functions. Fatty acid release from the membranes after pathogen encounter triggers the defense response (Savchenko et al., 2010). Linolenic acid (18:3) is a precursor for the production of the major cellular signaling components JA and oxylipins (Reinbothe et al., 2009). A reduction of the levels of oleic acid (18:1) triggers constitutive defense responses that are independent of SA signaling (Kachroo et al., 2001), but dependent on NO production (Mandal et al., 2012). Fatty acid homeostasis is disturbed under abiotic stress, as membrane composition changes are vital for the maintenance of membrane rigidity and functionality. Dehydration stress is shown to result in a reduction in 18:3 and increase in 18:1 lipid levels (Upchurch, 2008), and increased 18:3 levels by FAD3 or FAD8 overexpression enhanced drought tolerance in tobacco (Zhang et al., 2005). Manipulation of fatty acid composition can provide further insight into their function under stress combination.

#### Transcription factors

Regulatory modules like MAPKs-based pathways and core hormone signaling modules control the expression of a vast number of genes and therefore their manipulation in most cases have severe pleiotropic effects. Identification of downstream regulators involved in abiotic and biotic stress crosstalk such as TFs is important for more targeted manipulation and adaptation of plants to multiple stresses. The appropriate fine-tuning of their expression is an important aspect toward translation of scientific knowledge in crop plant improvement (Kasuga et al., 2004).

Bioinformatics and functional analyses have demonstrated that TFs involved in stress crosstalk comprise a diverse collection of members of the largest TF families in plants, such as NAC, MYB, AP2/ERF, WRKY, and others, reflecting the complexity of the genetic regulatory networks underlying stress crosstalk (Atkinson and Urwin, 2012; Shaik and Ramakrishna, 2014). Many members of these families are involved in regulation of leaf senescence, an integral component of both abiotic and biotic stress (Breeze et al., 2011). Moreover, in most cases the TFs identified are stress hormone-regulated, and therefore potentially act as molecular switches for the fine-tuning of hormonal responses.

Characterization of the mechanism of action of the candidate TFs involved in stress crosstalk is of great importance. For example, a TF with positive contribution to both abiotic and biotic stress tolerance can be directly useful for breeding combined stress tolerance. Functional characterization of several TFs has revealed various members that confer both abiotic and biotic stress tolerance. Overexpression of the rice OsNAC6 conferred tolerance to salt and dehydration stress as well as resistance to blast disease (Nakashima et al., 2007). Similarly in wheat, overexpression of the R2R3MYB gene TaPIMP1 results in drought stress tolerance and resistance to *Bipolaris sorokiniana* through increased expression of abiotic stress (many of them ABA inducible) and defense-related genes (Zhang et al., 2012). Members of the AP2/ERF TF family have been shown to be positive regulators of both abiotic and biotic stress (Jung et al., 2007; Zhang et al., 2009). DREB TFs are also members of the AP2/ERF family and important contributors to abiotic stress tolerance (Liu et al., 2013a) that may have additional signaling functions for biotic stress tolerance. AtDREB2A was upregulated in plants overexpressing the CC-NB-LRR gene ADR1 which conferred pathogen resistance and drought tolerance (Chini et al., 2004). Overexpression of OsDREB1B in tobacco resulted in increased resistance to abiotic stress and also virus infection (Gutha and Reddy, 2008).

Overexpression of AtHSFA1b provided stress hormone independent, but H_2_O_2_ signaling dependent increased tolerance to drought and resistance to bacterial and oomycete pathogens (Bechtold et al., 2013). It appears that the HSF TF gene family has broad biological functions in ROS signaling and defense responses and SAR regulation (Miller et al., 2008; Pick et al., 2012), which can be further exploited for building broad stress tolerance into crops. Whole genome expression meta analyses can provide evidence of potential antagonistic regulation in different stress responses for a given TF, by analyzing expression patterns under different stress conditions (Shaik and Ramakrishna, 2014). Detailed characterization of spatiotemporal expression and *cis*-element binding patterns is, however, required for the understanding of the underlying mode of regulation. This was recently elegantly demonstrated in the characterization of OsWRKY13 which exhibits tissue specific expression and condition specific binding to *cis*-elements of downstream genes and thereby inversely regulated resistance to drought and bacterial infection of rice (Xiao et al., 2013).

Functional conservation of TF functions across species can be exploited to take advantage of the wealth of experimental data generated in the model plant *Arabidopsis thaliana*. For example, the *Arabidopsis* AtBOS1, an R2R3MYB TF, as well as its homolog in tomato SlAIM1 appear to regulate tolerance to abiotic and biotic stress in the same way, as mutant plants exhibit reduced tolerance to salt stress as well as to *Botrytis* infection (Mengiste et al., 2003; Abuqamar et al., 2009). Further similar efforts should be undertaken to accelerate the translation of experimental observations obtained in model plants species to crops.

The results obtained by the functional characterization of TFs are encouraging as many of them appear to regulate cross-resistance in a unidirectional manner, in contrast to the observations at the level of hormonal regulation that point to antagonistic relationships. Therefore, their manipulation offers many opportunities to bypass the antagonistic effects on abiotic and biotic stress tolerance observed in the more upstream regulatory nodes.

#### Epigenetic modifications

Epigenetic modifications such as DNA cytosine methylation and histone residues methylation and acetylation contribute to the transcriptional control of amongst others adaptive responses to environmental stimuli (Mirouze and Paszkowski, 2011). A significant portion of these modifications appears to be persistent across generations and significantly contributes to phenotypic variation (Johannes et al., 2009). While cytosine methylation generally has repressive effects on gene transcription, leading to gene silencing, histone modifications can lead to transcriptional activation through local chromatin de-condensation which facilitates the accessibility of TFs (Liu et al., 2010). Recently, epigenetic modifications and specifically chromatin-regulated gene activation have been proposed to govern priming responses (Conrath, 2011). Genome wide approaches studying DNA methylation under abiotic and biotic stress have demonstrated widespread methylation alterations (Bilichak et al., 2012; Dowen et al., 2012). It would be of particular interest to further examine the occurrence of differential alterations and their impact under combinatorial stress.

Functional studies of chromatin remodeling enzymes have revealed a functional involvement of these enzymes in the regulation of both abiotic and biotic stress responses. Histone deacetylase 19 (HDA19) mutants exhibit enhanced basal expression of many SA-responsive genes (Kim et al., 2008) but decreased expression of ABA and JA/ET-responsive genes, and the mutants are hypersensitive to salt stress (Chen et al., 2010b). The histone lysine methyltransferase ATX1 is likely to be involved in dehydration stress signaling, as atx1 mutants were sensitive to drought and ATX1 methyltransferase activity positively regulated the expression of the ABA biosynthesis enzyme NCED3 (Ding et al., 2011). Interestingly, down-regulation of the TF WRKY70 during dehydration stress coincided with decreased presence of ATX1 at the WRKY70 gene locus (Ndamukong et al., 2010).

Chromatin structure can also be altered by the active deposition of variants of the canonical histones. Deposition of one of the these variants, H2A.Z, is linked to transcriptional activation in response to environmental stimuli (Coleman-Derr and Zilberman, 2012), and disruption of this mechanism leads to misregulated responses to both pathogens and elevated temperature (March-Diaz et al., 2008; Kumar and Wigge, 2010).

It would be highly interesting to investigate how a previously imposed stress predisposes plants at the methylation and chromatin level for the encounter of a subsequent stress, (de)sensitizing subsequent responses. This type of acclimation/predisposition may even be a useful tool for preparing seeds and propagated material for stressful environments.

#### R-gene resistance and systemic acquired resistance

The plant immune system consists of successive layers counteracting suppression of defense responses by pathogens through secretion of effector proteins (Hemetsberger et al., 2012). Recognition of the effectors by corresponding R-genes belonging to NB-LRR protein family or the effect of effectors on intracellular host proteins (guarded proteins) results in effector-triggered immunity (ETI). This is usually but not always manifested by localized cell death, termed the hypersensitivity response (Coll et al., 2011). The complexity in the regulation of ETI is outlined by network analyses of individual and combined hormone mutants, which revealed compensatory interactions in contrast to synergistic interaction observed in PTI (PAMP-triggered immunity; Tsuda et al., 2009), and which may explain the robustness of ETI to genetic perturbations. This robustness may be ideal in building tolerance to combinatorial stress through pyramiding R-genes with genes conferring abiotic stress tolerance.

However, it is becoming clear that there are multiple aspects of regulation at the NB-LRR protein level that are indispensable for the deployment of R-gene resistance (Heidrich et al., 2012). These include spatial regulation of NB-LRR accumulation in cellular compartments (e.g., the nucleus). Reduction of nuclear NB-LRR accumulation was shown to be responsible for the heat stress attenuation of disease resistance conferred by the proteins SNC1 and RPS4 in *Arabidopsis* (Zhu et al., 2010; Mang et al., 2012). Interestingly, mutants with reduced sensitivity to heat-induced defense inhibition were found to be based on changes in among others ABA biosynthesis enzymes, indicating that abiotic stress factors may affect R-gene compartmentation through ABA biosynthesis and signaling, although no further evidence is available. In addition, chaperone-mediated transport and folding of NB-LRR protein is important for their activity (Hubert et al., 2009). The heat shock protein HSP90 is a component of this chaperone machinery. HSP90 is also required for the maintenance of folding of other proteins under stress conditions (Wang et al., 2004), and could potentially become limiting for proper R-gene signaling or stress protection under combined stress conditions. The recent discovery that NB-LRR protein accumulation is controlled by microRNAs (Zhai et al., 2011) adds a novel layer of regulation that would be interesting to investigate under different stress conditions (Kulcheski et al., 2011).

Initial pathogen perception and interception through PTI or ETI triggers systemic signals that prime plant defense responses to effectively counter subsequent infection attempts and limit spreading of the disease. This is referred to as SAR. Many compounds and genes have been identified that function in mobile signal generation and transport. Conversion of MeSA produced at the infection site to SA at the systemic tissues appears to be a prerequisite for SAR manifestation (Park et al., 2007). Additional metabolites such as pipecolic acid, dehydroabietinal, azelaic acid, and glyceraldehyde 3-phosphate probably function in the amplification of the signal, with no clear conclusions yet on their precise placement in the SAR circuit pathway (Dempsey and Klessig, 2012). SAR has been shown to be affected by environmental conditions such as exposure to light (Griebel and Zeier, 2008) and abiotic stresses such as salinity, through ABA suppression of SA biosynthesis (Yasuda et al., 2008). The further investigation of the patterns of accumulation and transport of these metabolites under conditions of combined abiotic and biotic stress may reveal potential connections between their regulation and plant phenotypic responses to combined stress.

## APPROACHES FOR GENE IDENTIFICATION AND BREEDING FOR TOLERANCE TO STRESS COMBINATION

In accordance with individual abiotic and biotic stressors, each abiotic stress/pathogen/host combination should be treated independently as, despite the potential universal applicability of some interactions that were characterized in *Arabidopsis*, many unique interactions may be crucial for the phenotypic response. As a result improving crops to these complex stress conditions first requires an extensive phenotypic characterization at different levels of cellular regulation, i.e., transcription, translation, post-translation, and metabolites, as well as at different stages of plant development. As evidence from research on individual abiotic and biotic stress responses points to a strong dependency on developmental (Skirycz et al., 2010) as well as environmental factors (Luna et al., 2011), the environmental conditions and developmental stages of the plants should be appropriately defined before any interpretation of the phenotypic and molecular response can be done. Finally the different layers of defense can be differentially affected by abiotic stress imposition (**Figure 2**); therefore, the outcome of the interaction will vary with the defense mechanisms employed and on the pathogens involved.

Breeding for resistance to combinatorial stress is challenging. However, various novel approaches can aid in dissecting interactions between various types of stressors and identifying genetic components that can be breeding targets. The combination of different ~omics technologies has enabled the molecular dissection of plant phenotypes (Baerenfaller et al., 2012; Nagano et al., 2012). They provide information about the biological function of the whole gene set of an organism, and overlapping expression patterns might imply participation in common pathways (Quackenbush, 2003), enabling more efficient reverse genetic approaches. Utilization of‘~omics in combination with forward genetic approaches like association mapping (Chan et al., 2011) may narrow down the candidate genes responsible for the observed phenotypes and provide targets for functional characterization, further manipulation and improvement of crops through breeding. As mentioned previously, currently there are limited studies on the ~omics characterization of combined abiotic and biotic stress tolerance, however, functional characterization of differentially regulated genes is starting to provide interesting candidates for combined stress tolerance and their mode of action (Atkinson et al., 2013).

Manipulations that induce resistance to abiotic and biotic stress such as application of priming chemicals, followed by comprehensive phenotypic characterization can be used for candidate gene identification and molecular processes underlying stress cross-tolerance. Utilization of pre-existing chemical libraries for compounds that can prime abiotic and/or biotic stress tolerance and identification of their mode of action through chemical genetics approaches can both provide biotechnological targets for crop stress improvement and an opportunity to directly use the identified chemical in agricultural practice if no unintended side effects are observed (Hicks and Raikhel, 2009; McCourt and Desveaux, 2010; Okamoto et al., 2013). Moreover as the effects of chemical priming are shown to, in part, be exerted through induction of phosphorylation and other post-translational modifications (Beckers et al., 2009), probing these modifications and genetically manipulating the underlying codons to constitutively mimic them (Riano-Pachon et al., 2010) can result in altered responses under combinatorial stress.

Breeding for resistance to exposure to combined abiotic and biotic stress by incorporation of genetic components regulating the response to both stresses faces various challenges. For example, TFs can have thousands of binding sites across the genome (Lu et al., 2013), increasing the chance of unwanted pleiotropic effects and therefore more sophisticated deployment should be employed. Both expression regulation and binding specificity can be altered through promoter and binding domain engineering (Desai et al., 2009; Cox et al., 2013) which can be aided by comparative genomic approaches (Korkuc et al., 2014) and applied through novel site-specific mutagenesis techniques (Liu et al., 2013b). As selective and stimulus specific TF binding drives stress responses regulation (Xiao et al., 2013), implementation of the above methods will aid to fine-tune downstream targets toward the desired phenotypic response. A potential drawback of TF utilization is that resistance typically achieved by this approach is partial, and potentially prone to numerous antagonistic effects between stresses that cannot be predicted and can hinder efficient deployment for crop improvement to combined stresses.

Pyramiding genes that provide increased tolerance to either stress and do not (negatively) interact with each other offers an alternative route. Strong resistance mediated by R-genes, that appear to be robust to perturbations, can be pyramided with well-characterized genes conferring abiotic stress tolerance (Hu and Xiong, 2013; Kissoudis et al., unpublished data). R-gene robustness can be assessed by testing resistance responses under different abiotic stressors prior to pyramiding. The drawback of this approach is the quick breakdown of resistance due to evolving pathogens, and the fact that necrotrophic fungi resistance cannot be acquired with these genes. R-gene stacking aided by novel biotechnological approaches can reduce the risk of breakdown of R-gene-mediated resistance.

Pre-invasion defense mechanisms can be exploited, especially the one that is conferred by preformed or inducible physical barriers such as callose and antimicrobial compound deposition at the site of attempted penetration. As discussed earlier, callose deposition appears to be positively regulated by ABA signaling, therefore positive or no interaction should be expected under abiotic stress. Genes such as the OCP3 TF can be utilized, and for instance pyramiding abiotic stress tolerance with resistance conferred by *mlo* loss of function which sensitizes callose deposition at the site of infection for resistance against powdery mildew (Buschges et al., 1997) may be a viable route (Kissoudis et al. unpublished data). However, pleiotropic effects reported in *mlo* mutants such as compromised resistance against necrotrophic pathogens (Kumar et al., 2001) and accelerated senescence (Piffanelli et al., 2002) can have adverse consequences under stress combination.

The mechanisms through which abiotic stress tolerance is conferred can have a differential effect on disease resistance. As mentioned earlier, drought tolerance through ABA upregulation at the whole-plant level is expected to have antagonistic effects with SA signaling and therefore compromises resistance to biotrophs. Localized ABA sensitization in stomata (Bauer et al., 2013) can overcome these drawbacks and offer an advantage for resistance against pathogen that infect through stomata. Manipulation of developmental traits such as root system architecture can be beneficial for drought tolerance (Uga et al., 2013) with potentially no adverse effects on disease resistance, as they employ cell type specific signaling. Deployment of genes that have a protective function on proteins and cellular components under abiotic stress, such as dehydrins, LEA proteins or RNA chaperones (Kang et al., 2013) that apparently are downstream components of abiotic stress adaptation and mostly function through their structural properties, can minimize interaction with biotic stress signaling. Moreover, under salt stress, increased tolerance through Na^+^ compartmentalization in the vacuoles may offer an advantage in comparison with Na^+^ exclusion, as Na^+^ at high concentrations can have adverse effects on pathogen feeding and development.

Approaches that result in greater antioxidant capacity such as the accumulation of flavonoids appear to confer resistance to abiotic and oxidative stress (Nakabayashi et al., 2014) while overproduction of their derivatives, anthocyanins, increase resistance to the necrotrophic pathogen *B. cinerea* in tomato by minimizing ROS burst (Zhang et al., 2013). Therefore engineering for increased flavonoid accumulation can be promising in conferring resistance to multiple stressors, however, it is unknown how it can affect the deployment of hypersensitivity response due to disturbed ROS homeostasis and thus resistance against biotrophic pathogens.

**FIGURE 3:**
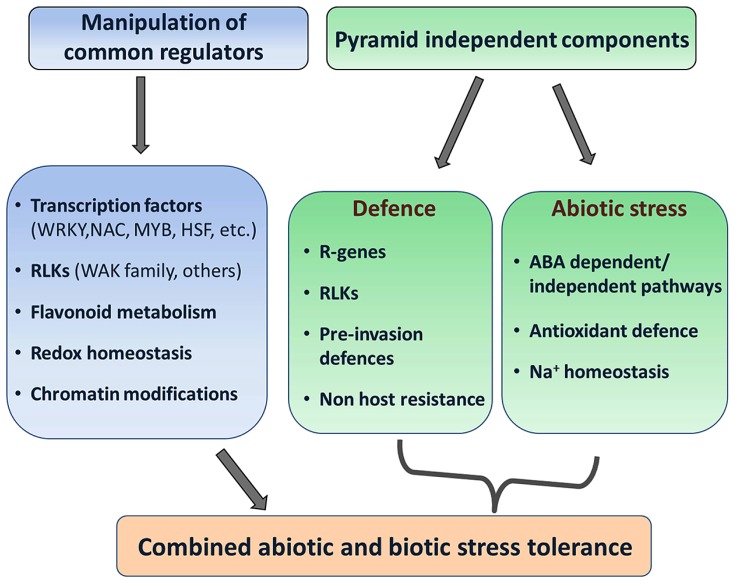
**Approaches for building combined abiotic and biotic stress tolerance in plants**. Two strategies are proposed through either the manipulation of genetic components which potentially regulate resistance to both stresses in a preferentially unidirectional manner, or the pyramiding of genes that independently confer abiotic or biotic stress resistance and do not (negatively) interact. The selection of individual components might differ depending on the pathogen and the abiotic stress scenario.

Exploitation and deployment of different strategies (**Figure 3**) under different abiotic stress/pathogen combinations will demonstrate their feasibility and applicability, further leading toward the goal of breeding for crops that maintain their robustness and yield performance under diverse environmental conditions.

## Conflict of Interest Statement

The authors declare that the research was conducted in the absence of any commercial or financial relationships that could be construed as a potential conflict of interest.
